# Downregulation of Protein Kinase CK2 Activity Facilitates Tumor Necrosis Factor-α-Mediated Chondrocyte Death through Apoptosis and Autophagy

**DOI:** 10.1371/journal.pone.0019163

**Published:** 2011-04-29

**Authors:** Sung Won Lee, Yeon Suk Song, Sang Yeob Lee, Young Geol Yoon, Sang Hwa Lee, Bong Soo Park, Il Yun, Hyantae Choi, Kunhong Kim, Won Tae Chung, Young Hyun Yoo

**Affiliations:** 1 Department of Rheumatology, Dong-A University College of Medicine, Busan, Korea; 2 Department of Anatomy and Cell Biology and Mitochondria Hub Regulation Center, Dong-A University College of Medicine, Busan, Korea; 3 Department of Microbiology, Dong-A University College of Medicine, Busan, Korea; 4 Department of Oral Anatomy and Cell Biology, Pusan National University College of Dentistry, Yangsan, Korea; 5 Department of Biochemistry and Molecular Biology and Center for Chronic Metabolic Disease Research, Yonsei University College of Medicine, Seoul, Korea; University of Western Ontario, Canada

## Abstract

Despite the numerous studies of protein kinase CK2, little progress has been made in understanding its function in chondrocyte death. Our previous study first demonstrated that CK2 is involved in apoptosis of rat articular chondrocytes. Recent studies have suggested that CK2 downregulation is associated with aging. Thus examining the involvement of CK2 downregulation in chondrocyte death is an urgently required task. We undertook this study to examine whether CK2 downregulation modulates chondrocyte death. We first measured CK2 activity in articular chondrocytes of 6-, 21- and 30-month-old rats. Noticeably, CK2 activity was downregulated in chondrocytes with advancing age. To build an *in vitro* experimental system for simulating tumor necrosis factor (TNF)-α-induced cell death in aged chondrocytes with decreased CK2 activity, chondrocytes were co-treated with CK2 inhibitors and TNF-α. Viability assay demonstrated that CK2 inhibitors facilitated TNF-α-mediated chondrocyte death. Pulsed-field gel electrophoresis, nuclear staining, flow cytometry, TUNEL staining, confocal microscopy, western blot and transmission electron microscopy were conducted to assess cell death modes. The results of multiple assays showed that this cell death was mediated by apoptosis. Importantly, autophagy was also involved in this process, as supported by the appearance of a punctuate LC3 pattern and autophagic vacuoles. The inhibition of autophagy by silencing of autophage-related genes 5 and 7 as well as by 3-methyladenine treatment protected chondrocytes against cell death and caspase activation, indicating that autophagy led to the induction of apoptosis. Autophagic cells were observed in cartilage obtained from osteoarthritis (OA) model rats and human OA patients. Our findings indicate that CK2 down regulation facilitates TNF-α-mediated chondrocyte death through apoptosis and autophagy. It should be clarified in the future if autophagy observed is a consequence versus a cause of the degeneration *in vivo.*

## Introduction

Osteoarthritis (OA) is characterized by the destruction of extracellular matrix and the loss of chondrocyte function [1]. Chondrocyte depletion was found to be a persistent and important event in OA. Mechanical injury, loss of extracellular matrix, loss of growth factors or excessive reactive oxygen species can induce chondrocyte depletion [2]. Because articular chondrocytes are solely responsible for the production and maintenance of the extracellular matrix, chondrocyte depletion is implicated in cartilage degeneration, which pertains to OA pathogenesis [3], [4]. Because apoptosis was believed to be a major cause of such cell depletion, most previous studies examining chondrocyte depletion during OA progression had focused on chondrocyte apoptosis. A variety of stimuli, such as nitric oxide (NO) [5], prostaglandin E2 [6], Fas ligand [7], tumor necrosis factor (TNF)-α [8], and TNF-related apoptosis-inducing ligand (TRAIL) [9] have been reported to induce apoptosis in chondrocytes. Enhanced chondrocyte apoptosis is now considered to be a sign of progressive cartilage joint degeneration in OA. However, an important question regarding the extent of the contribution of apoptotic cell death to chondrocyte depletion during OA progression remains unresolved. Several studies support the idea that another type of cell death, necrosis, can be involved in chondrocyte death during OA progression [2].

The role of cytokines in OA has been studied extensively. Among the inflammatory mediators associated with joint diseases, TNF-α has been established as a key mediator in the progression of cartilage degeneration. TNF-α promotes the further expression of cytokines and chemokines in synovial cells and chondrocytes, thereby maintaining the renewal of local inflammatory mediators [10], [11]. The presence of TNF-α correlates with a general loss of cartilage matrix molecules, such as type II collagen and aggrecan, due to the increased production of matrix metalloproteinases and synthesis of matrix molecules [12].

TNF-α induces apoptosis in chondrocytes by two mechanisms: direct induction of apoptosis and indirect priming of cells for apoptosis by Fas ligand presentation [8], [13]. The Bcl-2/Bax family of proto-oncogenes is known to involve in the cellular signaling pathways of chondrocyte apoptosis induced by TNF-α. A transcription factor NF-κB is also involved in this process [14]. These pathways lead to the activation of effector caspases (such as caspase-3) that cleave cellular proteins. During apoptosis, caspases target housekeeping, structural and cytoskeletal proteins and activate inhibitors of caspase-activated deoxyribonuclease or poly (ADP-ribose) polymerase (PARP) [15].

Protein kinase (PK) CK2, which is ubiquitously distributed in the cytoplasm and nuclei of eukaryotic cells, is a messenger-independent protein serine/threonine kinase [16], [17]. Previous studies have indicated that CK2 participates in a series of complex cellular functions, including cell growth and proliferation, by catalyzing the phosphorylation of a large number of proteins [18]. CK2 also participates in the regulation of apoptosis by phosphorylating some apoptosis-related factors [18]–[21]. We previously demonstrated that CK2 is involved in NO-induced apoptosis of rat articular chondrocytes [22]. Despite the numerous studies of CK2, however, little progress has been made in understanding its function in chondrocytes.

Recent studies have suggested that CK2 downregulation is associated with aging. One study demonstrated that downregulation of CK2 activity is tightly associated with cellular senescence as well as with organismal aging [23]. In addition, CK2 was shown to play a critical role in regulating cytoskeletal reorganization during senescence progression [24]. It was also reported that silencing of the CK2α and CK2α' genes during cellular senescence is mediated by DNA methylation [25]

This study was undertaken to examine whether CK2 downregulation is involved in OA pathogenesis. As shown by our results, CK2 activity is downregulated in chondrocytes of aged articular cartilage, and the inhibition of CK2 activity by CK2 inhibitor treatment sensitizes TNF-α mediated chondrocyte death *in vitro* through apoptosis and autophagy. In addition, we observed puncta formation of microtubule-associated protein 1 light chain 3 (LC3) in chondrocytes from cartilage obtained from both human OA patients and rats with surgically induced OA, indicating that autophagy is involved in OA pathogenesis.

## Materials and Methods

### Ethics statement

The animal protocol used in CK2 activity assay was reviewed and approved by the Pusan National University-Institutional Animal Care and Use Committee (PNU-2008-0008) under their ethical procedures and scientific care. The animal protocols used in cell culture and rat OA model were reviewed and approved by the Dong-A University-Institutional Animal Care and Use Committee under their ethical procedures and scientific care (DIACUC-07-8). The study using Human samples was reviewed and approved by the Dong-A University Hospital Institutional Review Board (DUHIRB-10-10-23). Written informed consent was obtained from all participants.

### Reagents

The following reagents were obtained commercially: polyclonal rabbit anti-human caspase-2L and caspase-8 from Santa Cruz Biotechnology (Santa Cruz, CA); caspase-3 antibody and HRP-conjugated goat anti-rabbit IgGs from Cell Signalling Technology (Beverly, MA); LC3 antibody from Novus Biologicals (Littleton, CO); ATG5 and ATG7 antibodies from Abonova (Taipei, Taiwan); FITC-conjugated goat anti-rabbit IgGs from Vector (Burlingame, CA); TNF-α and ApopTag Red In Situ Apoptosis Detection Kit from Millipore (Temecular, CA); Dulbecco's Modified Eagle's Medium (DMEM) and fetal bovine calf serum (FBS) from Gibco (Gaithersburg, MD); Lysotracker from Invitrogen (Carlsbad, CA); DRAQ5TM from Axxora (San Diego, CA); β-actin antibody, dimethyl sulfoxide (DMSO), RNase A, proteinase K, protease inhibitor cocktail, propidium iodide (PI), 3-methyladenine (3MA), bafilomycin A1, 5,6-dichlorobenzimidazol riboside (DRB), apigenin, 4,5,6,7-tetrabromobenzotriazole (TBB) and type II collagenase from Sigma (St. Louis, MO); Super Signal West Pico Chemiluminescent Substrate from Pierce (Rockford, IL).

### CK2 activity assay

Specific pathogen-free male Sprague Dawley rats were obtained from Samtako (Osan, Korea). Animals were housed individually in polycarbonate cages with wood chip bedding and were maintained in an air-conditioned animal room (temperature: 24°C, relative humidity: 55±5%) on a 12-hr light/dark cycle at Pusan National University for 7 days. The rats (6, 21 and 30 months of age) were killed and the knee joint cartilage was used for the CK2 activity assay. Three animals were used from each age group. CK2 activity was measured as previously described with slight modification [26]. Three micrograms of bacterially expressed GST-CS (CK2 substrate) protein were incubated with glutathione sepharose 4B beads (GE Healthcare) for 30 min, after which they were washed twice with 1× kinase buffer (4 mM MOPS, pH 7.2, 5 mM β-glycerol phosphate, 1 mM EGTA, 200 µM sodium orthovanadate and 200 µM dithiothreitol). The beads were then incubated with 100 µg of total cell lysate in 50 µl of kinase reaction buffer [10 µl of 5× kinase buffer and 10 µl of magnesium/ATP cocktail solution (90 µl of 75 mM MgCl_2_/500 mM ATP plus 10 µl (100 µCi) of [γ-^32^P]-ATP (3000 Ci/mmole)] for 30 min at 30°C. For experimental and negative controls, the beads were incubated with 50 ng recombinant active CK2 (Millipore) and without cell lysates, respectively. The reactions were stopped by washing twice with 1× kinase buffer. The samples were resuspended in 30 µl of 1× SDS loading buffer and subjected to SDS-PAGE and autoradiography. CK2 activity was quantified by counting the radioactivity of each excised GST-CS band using a β-counter.

### Cell culture of articular chondrocytes

Five-week-old male specific pathogen-free Sprague Dawley rats were obtained from Samtako (Osan, Korea). Rat articular chondrocytes for primary culture were isolated from knee joint cartilage slices by enzymatic digestion for 1 h with 0.2% type II collagenase (381 units/mg) in DMEM. After the isolated cells were collected by brief centrifugation, they were resuspended in DMEM supplemented with 10% (v/v) FBS, 50 µg/ml streptomycin and 50 units/ml penicillin (Gibco). The cells were plated on culture dishes at a density of 5×10^4^ cells/cm^2^. The medium was replaced every 2 days, and they reached confluence after approximately 5 days in culture.

### Treatment of TNF-α and pharmacological reagents

To induce cell death, chondrocytes from day 4 cultures were treated with TNF-α. To examine whether the inhibition of CK2 activity modulates the extent of TNF-α-mediated chondrocyte death, cells were incubated with one of three CK2 inhibitors (apigenin, DRB or TBB) in the presence or absence of TNF-α. To examine the effect of inhibition of autophagy on chondrocyte death, cells were pretreated with 3MA for 24 h and were further exposed to TNF-α in the presence or absence of the CK2 inhibitor DRB for 24 h.

### Cell viability assay

Cell viability was determined by the Vi-Cell (Beckman Coulter, CA) cell counter that performs an automated trypan blue exclusion assay.

### Nuclear morphology study for apoptosis

Cell suspensions were cytospun onto clean fat-free glass slides using a cytocentrifuge. Centrifuged samples were fixed for 10 min in 4% paraformaldehyde and stained in 10 µg/ml PI for 30 min at 4°C.

### DNA electrophoresis

Cells (2×10^6^) were resuspended in 1.5 ml of lysis buffer [10 mM Tris (pH 7.5), 10 mM EDTA (pH 8.0), 10 mM NaCl and 0.5% SDS] into which proteinase K (200 µg/ml) was added. After samples were incubated overnight at 48°C, 200 µl of ice cold 5 M NaCl was added and the supernatant containing fragmented DNA was collected after centrifugation. The DNA was then precipitated overnight at −20°C in 50% isopropanol and RNase A-treated for 1 h at 37°C. A loading buffer containing 100 mM EDTA, 0.5% SDS, 40% sucrose, and 0.05% bromophenol blue was added at 1:5 (v/v). Separation was achieved in 2% agarose gels in Tris-Acetic acid/EDTA buffer (containing 0.5 µg/ml ethidium bromide) using 50 mA for 1.5 h.

### Pulsed-field gel electrophoresis (PFGE)

Cells (2×10^6^) were suspended in 50 µl of PBS containing 1% low melting temperature agarose. The cell suspension was poured into a template (5×2×10 mm), plugged, and cooled on ice. The hardened agarose gel blocks were incubated with 250 µl of a mixture of proteinase K (1 mg/ml), N-lauroyl sarcosin sodium (1% w/v), and 0.5 M EDTA (pH 9.2) at 50°C for 48 h. After incubation, half the volume of the digested agarose gel block was loaded into a sample well of a 1% (w/v) agarose gel (Sigma type II, 150×150×4.4 mm) in 0.5× TBE buffer (89 mM Tris-boric acid, 2 mM EDTA, pH 8.0). PFGE was carried out in 0.5× TBE maintained at 14°C by circulating cool water for 16 h (constant, 6 V; switch times are initial 60 sec and final 90 sec), using the CHEF Mapper XA System from Bio-Rad. DNA in the gel was stained with ethidium bromide and detected with LAS-3000Plus (Fuji Photo Film Company, Kanagawa, Japan).

### Western blot analysis

Cells (2×10^6^) were washed twice with ice-cold PBS, resuspended in 200 µl ice-cold solubilizing buffer [300 mM NaCl, 50 mM Tris-Cl (pH 7.6), 0.5% TritonX-100, protease-inhibitor cocktail] and incubated at 4°C for 30 min. The lysates were centrifuged at 14,000 rpm for 20 min at 4°C. Protein concentrations of cell lysates were determined with Bradford protein assay reagent (Bio-Rad) and 40 µg of proteins were loaded onto 7.5–15% SDS/PAGE. The gels were transferred to nitrocellulose membrane (Amersham Pharmacia Biotech, Piscataway, NJ) and reacted with each antibody. Immunostaining with antibodies was performed using the Super Signal West Pico enhanced chemiluminescence substrate and detected with LAS-3000PLUS.

### Quantification of DNA hypoploidy and cell cycle phase analysis by flow cytometry

Ice-cold 95% ethanol with 0.5% Tween-20 was added to cell suspensions to a final concentration of 70% ethanol. Fixed cells were pelleted and washed with PBS containing 1% bovine serum albumin (BSA). Cells were resuspended in 1 ml PBS containing 11 Kunitz U/ml RNase, incubated at 4°C for 30 min, washed once with BSA-PBS and resuspended in PI solution (50 µg/ml). After the cells had been incubated at 4°C for 30 min in the dark and washed with PBS, DNA content was measured using an Epics XL (Beckman Coulter, FL), and the data were analyzed using the Multicycle software which allowed a simultaneous estimation of cell cycle parameters and apoptosis.

### Immunofluorescence staining, confocal microscopy and quantification

Cell suspensions were cytospun onto clean fat-free glass slides using a cytocentrifuge. Cells were incubated with an LC3 primary antibody for 1 h at 37°C, washed 3 times for 5 min each with PBS, incubated with a FITC-conjugated secondary antibody for 1 h at room temperature and counterstained with DRAQ5^TM^. Fluorescence images were observed and analyzed using a Zeiss LSM 510 laser-scanning confocal microscope (Gőettingen, Germany). To quantify those cells that showed a punctuate pattern, at least 300 cells from each experiment were counted by an observer who was blinded with regard to the experimental group.

### Transmission electron microscopy

Twenty four h after treatment, cells were harvested, pelleted and fixed in 2.5% glutaraldehyde in phosphate buffer. After being rinsed with phosphate buffer, the samples were postfixed in 1% osmium tetroxide for 1 h, rinsed with water, dehydrated in a graded series of ethanol followed by propylene oxide and kept overnight in Epon812. The samples were embedded in Epon812 and cured in an oven at 60°C. Ultrathin sections were obtained with a Reichert Ultracut E microtome. The sections were stained with uranyl acetate and lead citrate and observed using a transmission electron microscope (Hitachi). For each treatment or control group, at least 200 cells from randomly chosen transmission electron microscopy fields were observed.

### TUNEL staining of cell suspensions

Cell suspensions were cytospun onto clean fat-free glass slides in a cytocentrifuge. After being fixed with 4% paraformaldehyde, the cells were incubated with terminal deoxynucleotidyl transferase (TdT) enzyme for 1 h at 37°C, and antidigoxigenin-rhodamine was applied for 30 min at room temperature. Afterward the cells were incubated with an LC3 primary antibody for 1 h at 37°C. Nuclei were counterstained with DRAQ5^TM^. Fluorescent images were observed and analyzed using a Zeiss LSM 510 laser-scanning confocal microscope.

### Autophagy flux assay

To analyze the flux of autophagy induced by cotreatment with TNF-α and DRB cells were pretreated with bafilomycin for 24 h and were further exposed to TNF-α in the presence or absence of the CK2 inhibitor DRB for 24 h. Densitometric analysis of endogenous LC3-I and -II detected by Western blot assay was performed using Multi Gauge V2.1 (Fuji Photo Film Company, Kanagawa, Japan). Quantification of cells showing a punctuate pattern was performed described as above. Suppression by bafilomycin A1 of the development of acidic vesicular organelles was analyzed by flow cytometry. Cells were labeled by incubation with 0.75 µM LysoTracker in the culture medium at 37°C for 30 min. After incubation, they were washed with PBS and immediately analyzed by flow cytometry [excitation: 488 nm (argon laser), emission filter: 620 nm] using an Epics XL.

### Transfection of ATG5 and ATG7 small interfering RNA

The inhibition of autophagy was also carried out by knock-down of ATG-5 or ATG-7 genes. Using BLOCK-iT™ Fluorescent Oligo for electroporation (Invitrogen), we first optimized the conditions rat articular cartilage chondrocytes transfection and achieved >90% transfection efficiency using an electroporator (Neon™ transfection system, Invitrogen). ATG5 and ATG7 stealth siRNAs were designed based on the rat ATG-5 (NM_001014250.1) and ATG-7 (NM_001012097.1) cDNA reference sequences using the BLOCK-iT™ RNAi Designer (Invitrogen). ATG5 stealth siRNA were as follow: sense, 5′-AUC UCA UCC UGA UAG AGA GUA AAG C-3′; antisense, 5′-AAU AGU AUG GCU CUG CUU CUC GUU C-3′. ATG7 stealth siRNA were as follow: sense, 5′-AGA AGU AGC AGC CAA GCU UGU AUC C-3′; antisense, 5′-GGA UAC AAG CUU GGC UGC UAC UUC U-3′. As a negative control, stealth RNAi negative control (Invitrogen) was used. Chondrocytes were washed in PBS and resuspended in resuspension buffer R included with Neon™ kit (Invitrogen). ATG5 or ATG7 stealth siRNA and stealth RNAi negative control (each 200 nM) were electroporated twice into chondrocytes with pulse voltage of 1300V and pulse width of 20 ms using Neon™ transfection system. Eletroporated cells were resuspended in culture medium containing serum and supplements without antibiotics and incubated for 24 h at 37°C in a humidified 5% CO_2_ incubator. Cells were further exposed to TNF-α in the presence of DRB for 24 h.

### Rat OA model and human OA samples

#### Animals

Sprague-Dawley rats weighing 175–200 g were used obtained from Samtako (Osan, Korea). A total of nine rats were included in this group. The anterior cruciate ligament was transected using a cutting hook. Animals were maintained in their cages, where they moved freely without post-operative casts. After three days, they were trained in a treadmill exercise box for the next four weeks. Animals were then sacrificed. Both knee joints of each animal were dissected, and the femoral condyles were fixed in PBS (pH 7.4) containing 4% paraformaldehyde, decalcified in 12.5% EDTA, dehydrated and embedded in paraffin blocks. Non-operated rats were used as a control.

#### Human OA samples

Human articular cartilage samples were obtained from the knee joints of 25 patients (ages: 43–82 years; mean age: 68.4 years) during total knee replacement surgery due to OA. All patients attended the Rheumatology Clinic at Dong-A University Hospital (Busan, Korea). Full-thickness cartilage slices were taken from the medial femoral condyles above the subchondral bone. All patients were classified as radiographically grade IV OA based on the Kellgren/Lawrence (K/L) classification. In addition, healthy cartilage samples were obtained from the autopsies of two accident victims (ages 22 and 24 years old). Cartilage samples were fixed in PBS (pH 7.4) containing 4% paraformaldehyde, dehydrated and embedded in paraffin blocks. This study was reviewed and approved by the Dong-A University Hospital Institutional Review Board (DUHIRB-10-10-23). Written informed consent was obtained from all participants.

### Tissue preparation and LC3 immunostaining

Five-micrometer microsections were prepared. The sections were incubated with TdT enzyme for 1 h at 37°C, and antidigoxigenin-rhodamine was applied for 30 min at room temperature. Afterward the cells were incubated with an LC3 primary antibody for 1 h at 37°C. Fluorescent images were observed and photographed using a Zeiss LSM 510 laser-scanning confocal microscope under DIC optics without counterstaing.

### Statistical analysis

Four independent experiments were carried out *in vitro*. The results are expressed as means ± S.D. from four experiments, each performed in triplicate.

## Results

### Decrease in CK2 activity in chondrocytes of rat articular cartilage

**Figure 1:**
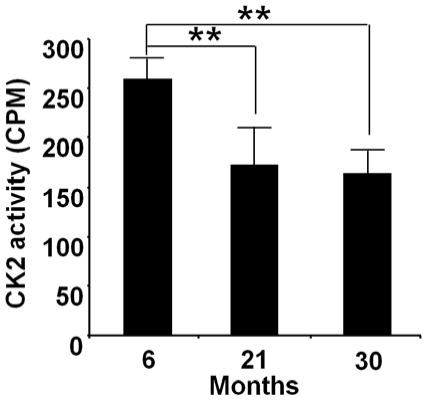
Decrease in CK2 activity in articular chondrocytes of aged rats. Lysates from articular chondrocytes of 6-, 21- and 30-month-old rats were used in kinase assays and quantified radioactivity determined using a β-counter. CK2 activity in the articular chondrocytes of 21- and 30-month-old rats was significantly decreased by 30% and 40%, respectively, compared to their 6-month-old counterparts (** *P*<0.01). Values are expressed as mean ± S.D.

CK2 activity was significantly decreased in rat articular cartilage chondrocytes with advancing age. CK2 activity in the articular chondrocytes of 21- and 30-month-old rats was decreased by 30% and 40%, respectively, compared to their 6-month-old counterparts (Fig. 1).

### CK2 inhibitors facilitate TNF-α-mediated chondrocyte death via apoptosis

**Figure 2:**
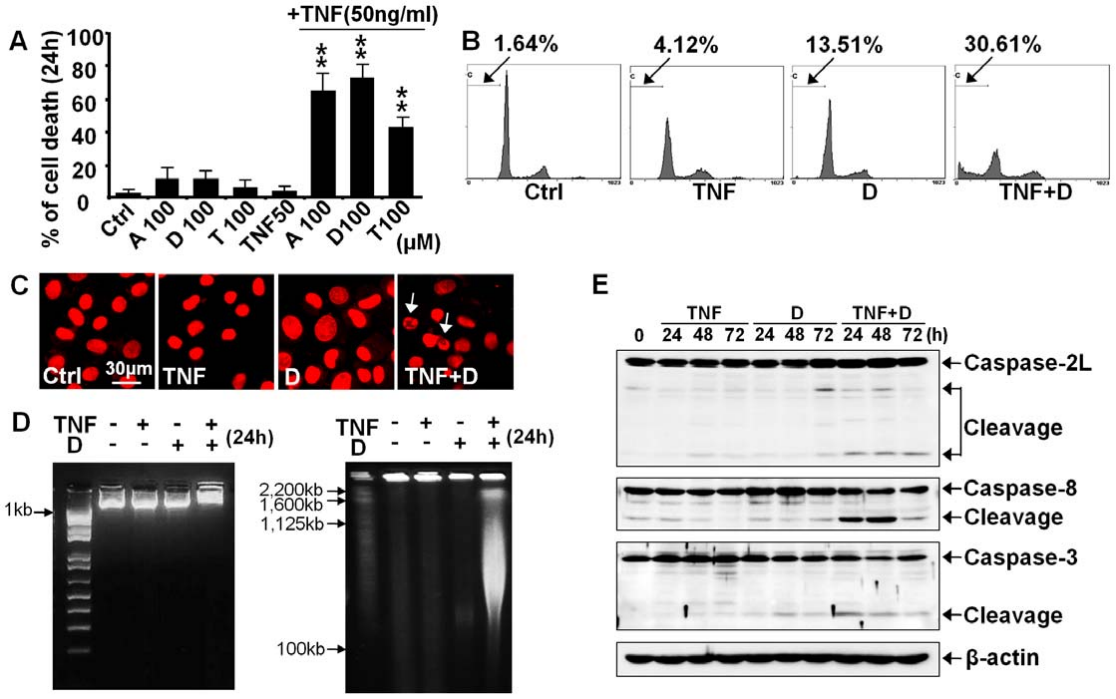
Facilitation of TNF-α-mediated chondrocyte death by CK2 inhibitors via apoptosis. To examine whether the inhibition of CK2 activity modulates the extent of TNF-α-mediated chondrocyte death, cells were co-treated with one of three CK2 inhibitors and TNF-α (50 ng/ml) for 24 h. Ctrl, control; A, apigenin; D, DRB; T, TBB; TNF, TNF-α. (A) Cells were harvested 24 h after treatment, and viability was determined using a cell counter performing an automated trypan blue exclusion assay. CK2 inhibitors significantly facilitated TNF-α-mediated chondrocyte death compared to TNF-α treatment alone (** *P*<0.01). Values are expressed as mean ± S.D. (B) Representative histograms showing the percentage of subdiploid apoptotic cells. DRB facilitated the TNF-α-induced accumulation of subdiploid apoptotic cells (arrows). (C) PI staining showed that DRB facilitated TNF-α-induced nuclear condensation. (D) DNA electrophoresis and PFGE. hough conventional DNA electrophoresis did not reveal a DNA ladder (left panel), PFGE revealed the disintegration of nuclear DNA into giant fragments of 1-2 Mbp and high molecular-weight fragments of 100-1000 Kbp in cells co-treated with DRB and TNF-α. (E) A western blot assay showed that DRB facilitated the TNF-α-induced degradation of caspase-2L, -8, and -3 subtypes and the production of caspase-2L, -8 and -3 cleaved products. For this assay, cells were harvested 24, 48 and 72 h after treatment. β-actin, a loading control.

To build an *in vitro* experimental system for simulating TNF-α-induced cell death in aged chondrocytes with decreased CK2 activity, we used CK2 inhibitors. To examine whether the inhibition of CK2 activity modulates the extent of TNF-α-mediated chondrocyte death, cells were co-treated with one of three CK2 inhibitors (apigenin, DRB or TBB) and TNF-α. The viability assay showed that treatment with CK2 inhibitors alone at concentrations of 75 and 100 µM led to a slight decrease in chondrocyte viability (Figure S1). Importantly, downregulation of CK2 activity by these inhibitors facilitated TNF-α-mediated chondrocyte death (Fig. 2A). Because the CK2 inhibitors significantly facilitated TNF-α-mediated chondrocyte death at 100 µM, this single concentration was used to further explore the mechanism underlying this process. At this dose, CK2 inhibitors decreased CK2 activity in chondrocytes to approximately half in comparison with the control cells. We first undertook various assays to test whether this cell death induction is mediated via apoptosis. Flow cytometry demonstrated the accumulation of subdiploid cells (Fig. 2B), and the nuclear morphology assay showed nuclear condensation (Fig. 2C). Although cells that were co-treated with DRB and TNF-α failed to show ladder-like DNA fragments from their genomic DNA on a standard agarose gel, PFGE revealed the disintegration of nuclear DNA into giant fragments of 1-2 Mbp and high molecular-weight fragments of 100–1000 Kbp (Fig. 2D). Considering that caspases were known to play essential roles in most types of apoptosis, we examined whether caspase subtypes were activated after co-treatment of DRB and TNF-α. A western blot assay showed that caspase-2L, -8, and -3 were activated in cells co-treated with DRB and TNF-α (Fig. 2E). These data support the idea that inhibition of CK2 activity facilitates TNF-α-mediated chondrocyte death via apoptosis. Another CK2 inhibitor, apigenin, showed similar effects of inducing TNF-α-mediated chondrocyte death (Figure S2). Silencing of CK2α by siRNA also facilitated TNF-α-mediated chondrocyte death (Figure S3A). Overexpression of CK2α reduced TNF-α-mediated chondrocyte death (Figure S3B).

### Autophagy is involved in facilitating the TNF-α-mediated chondrocyte death induced by CK2 inhibitors

**Figure 3:**
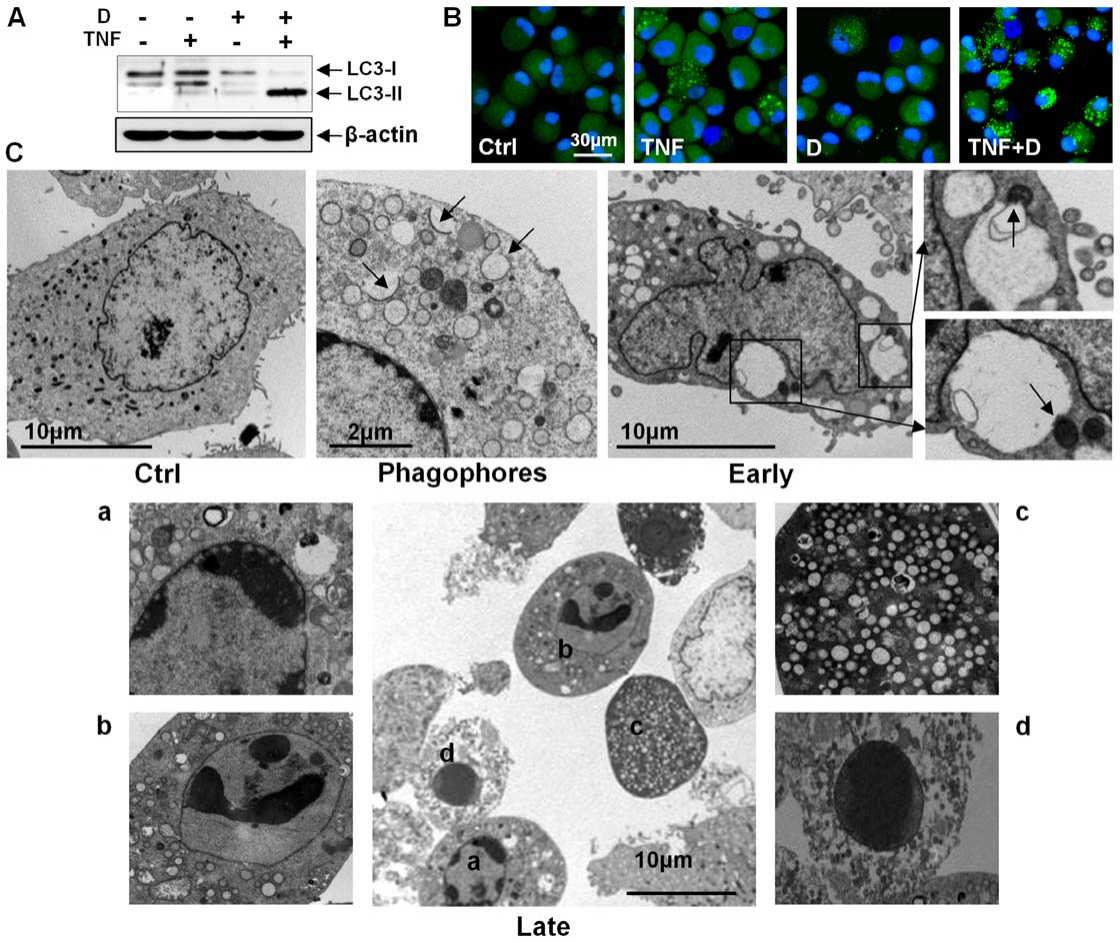
Autophagy is involved in the facilitation of TNF-α-mediated chondrocyte death by a CK2 inhibitor. For following assays, cells were harvested 24 h after treatment. (A) A western blot assay showed an increase of LC3-II levels in cells co-treated with DRB and TNF-α. β-actin, a loading control. (B) Immunofluorescence microscopy showed the appearance of a punctuate LC3 pattern in cells co-treated with DRB and TNF-α. (C) Tranmission electron microscopy. Representative data are shown. Upper left panel: untreated control. This control cell showed a normal distribution of organelles. Upper middle panel: the presence of phagopores (arrows) in cells co-treated with DRB and TNF-α. Upper right panel; early autophagic cell with a nucleus and microvilli that appeared normal. This cell had numerous autophagic vacuoles in the cytoplasm, large vacuoles approaching mitochondria (arrow) and membrane whorls. Lower panel: several cells with features of late-stage cell death. Cells were round and had no microvilli. Vacuoles contained phagocytosed cell fragments and myelin figures (cell a). Most cells with features of this late autophagic cell death showed peripheral chromatin condensation in their nuclei, a hallmark of apoptosis (cells a and b). In a highly vacuolated cell with features of very late cell death, all cell organelles had disappeared (cell c). Another cell showed features of very late cell death (cell d); its nucleus was highly condensed, and no cytoplasmic components, including vacuoles, were observable. See Figure 2 for other definitions.

We next tested whether another cell death mode, autophagy, was involved in this cell death induction. We first examined the conversion of LC3 from an 18-kDa form (LC3-I) to a faster-migrating 16-kDa form (LC3-II). A western blot assay demonstrated an increase in LC3-II levels in cells co-treated with DRB and TNF-α but not in cells treated with DRB or TNF-α alone (Fig. 3A). Fluorescence microscopy demonstrated that the number of cells showing a punctuate LC3 pattern, indicating LC3 aggregation, increased with co-treatment of DRB and TNF-α (Fig. 3B). We next used transmission electron microcopy to confirm the presence of autophagic vacuoles in cells co-treated with DRB and TNF-α (Fig. 3C). We observed the presence of phagophores in cells co-treated with DRB and TNF-α (Fig. 3C, phagophores). Early autophagic cells which had nuclei and microvilli with normal appearances had autophagic vacuoles (Fig. 3C, early). In these cells, some vacuoles were approaching mitochondria and contained membrane whorls. We also observed cells with features of late-stage cell death (Fig. 3C, late): those cells were round and had no microvilli, and their vacuoles contained phagocytosed cell fragments and myelin figures. Importantly, most cells showing features of this late autophagic cell death showed peripheral chromatin condensation in their nuclei, a hallmark of apoptosis. We also observed cells with features of later-stage cell death. In those highly vacuolated cells, all cell organelles had disappeared. We also observed cells with features of very late-stage cell death, in which all cytoplasmic components, including vacuoles, were degraded, and the nuclei were highly condensed.

### Prevention of lysosomal degradation by bafilomycin A1 enhanced the amount of LC3-II and the appearance of a punctuate LC3 pattern in chondrocytes co-treated with TNF-α and DRB

**Figure 4:**
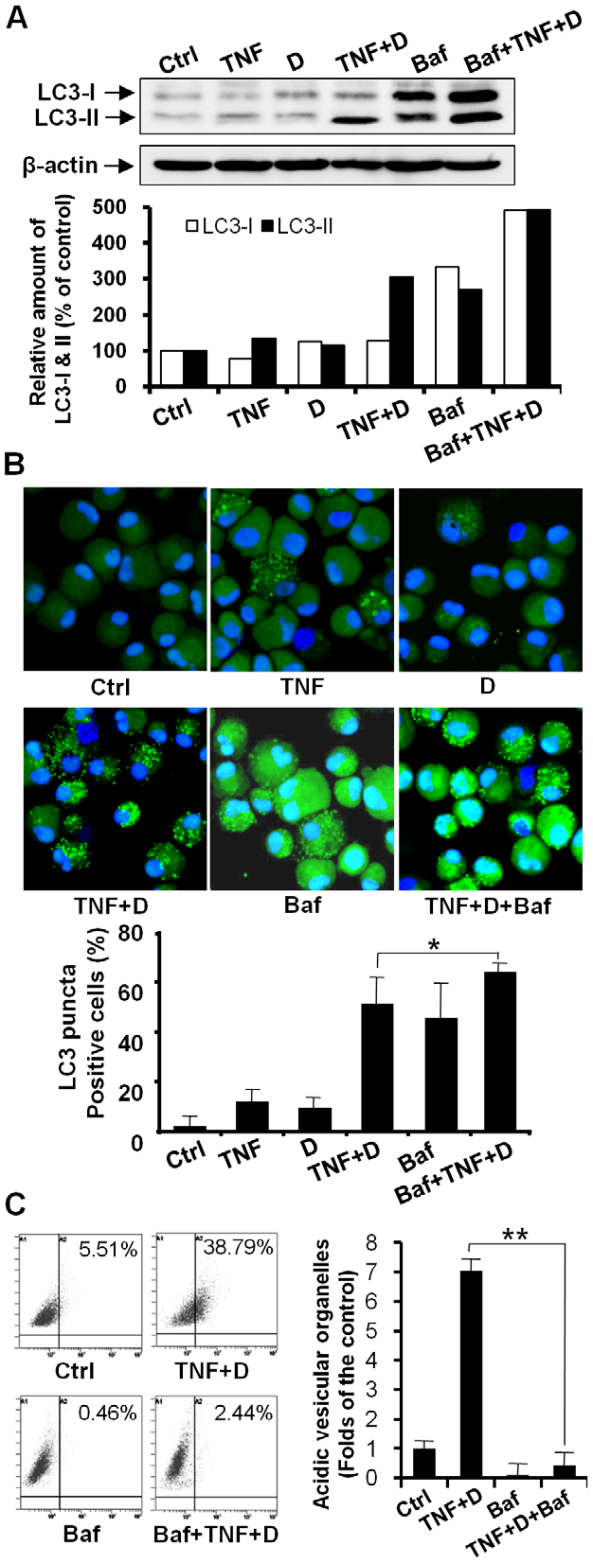
Prevention of lysosomal degradation by bafilomycin A1 enhanced the amount of LC3-II and the appearance of a punctuate LC3 pattern in chondrocytes co-treated with TNF-α and DRB. Cells were pretreated with bafilomycin A1 for 24 h and were further exposed to TNF-α in the presence or absence of the CK2 inhibitor DRB for 24 h. Baf, bafilomycin A1. (A) A western blot assay showed that an increase in LC3-II levels in cells co-treated with DRB and TNF-α was enhanced by bafilomycin A1 treatment. β-actin, a loading control. (B) Confocal microscopy and quantification assay demonstrated that the appearance of a punctuate LC3 pattern in chondrocytes co-treated with DRB and TNF-α was enhanced by bafilomycin A1 treatment (* *P*<0.05). (C) Flow cytometry demonstrated that bafilomycin A1 suppressed the development of acidic vesicular organelles. Bafilomycin A1 significantly decreased the cells having LysoTracker-positive vacuoles (** *P*<0.01). See Figure 2 for other definitions.

We further analyzed autophagy flux based on turnover of LC3-II by western blot in the presence and absence lysosomal degradation. To prevent lysosomal degradation, bafilomycin A1 was used [27], [28]. Considering that bafilomycin A1 is toxic to cells to some extent, we treated cells with 10 nM bafilomycin A1, the dose at which bafilomycin A1 did not yield any significant cellular damage after three days in culture. A western blot assay showed that an increase in LC3-II levels in cells co-treated with DRB and TNF-α was markedly enhanced by bafilomycin A1 treatment (Fig. 4A). Confocal microscopic observation and quantification assay revealed that the appearance of a punctuate LC3 pattern in chondrocytes co-treated with DRB and TNF-α was enhanced by bafilomycin A1 treatment (Fig. 4B). The observation and quantification of cells with LysoTracker-labeled organelles and flow cytometry analysis demonstrated that bafilomycin A1 suppressed the development of acidic vesicular organelles in co-treated cells (Fig. 4C).

### LC3 punta were detected in TUNEL positive cells

**Figure 5:**
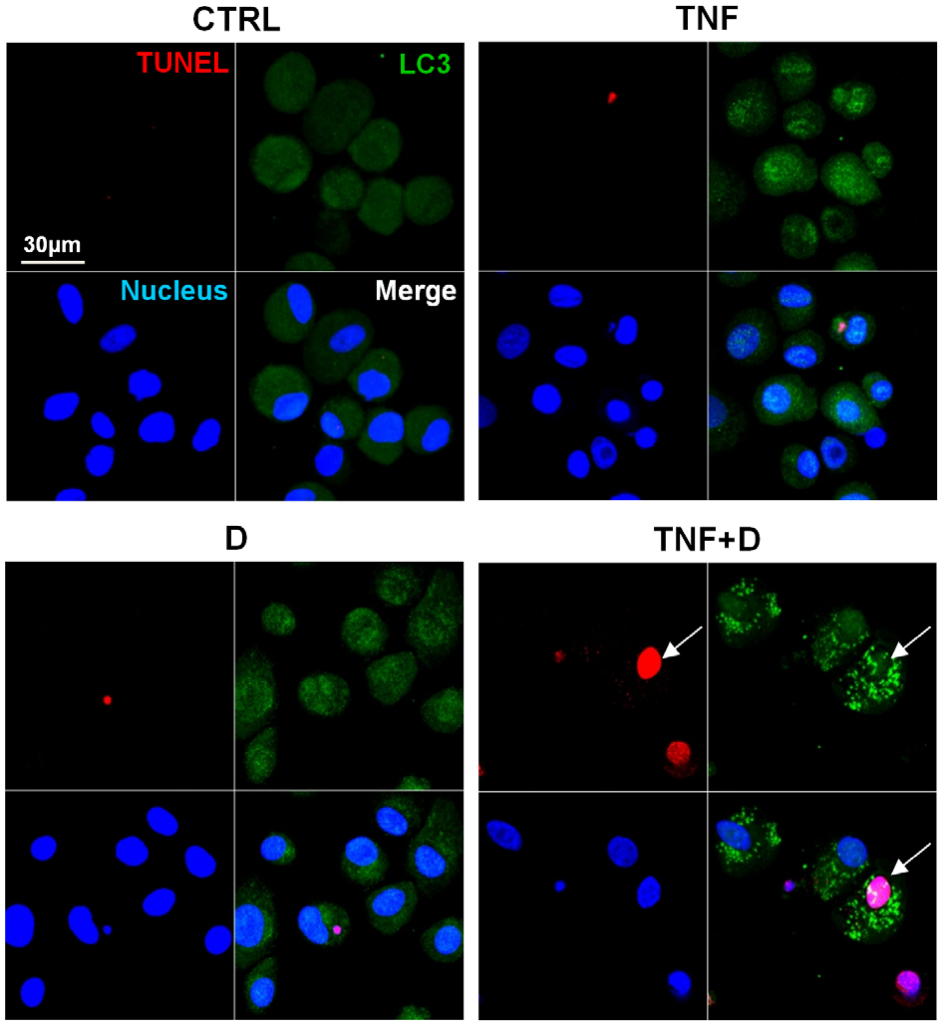
LC3 puncta were detected in TUNEL positive apoptotic cells. In a group co-treated with DRB and TNF-α for 24 h, a cell showing a punctuate LC3 pattern is TUNEL positive (arrow). See Figure 2 for other definitions.

We further examined whether that a punctuate LC3 pattern was observed in cells undergoing apoptosis. Noticeably, we observed a punctuate LC3 pattern in TUNEL positive cells (Fig. 5). Approximately 30% of cells showing LC3 punta were TUNEL positive.

### Inhibition of autophagy by 3MA or siRNA against autophagy-related genes protects chondrocytes against apoptosis as well as autophagy

**Figure 6:**
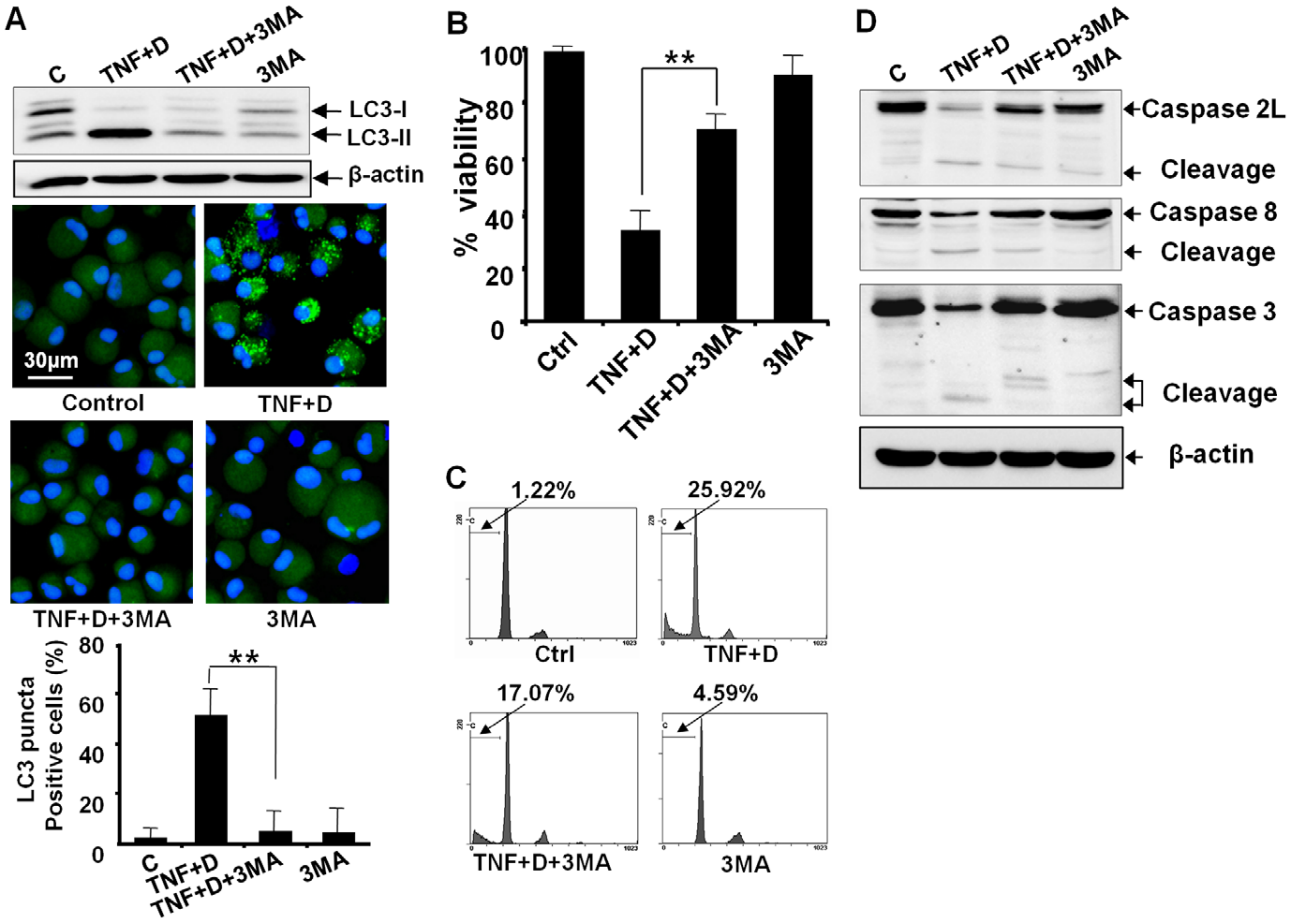
Inhibition of autophagy by 3MA protects chondrocytes against apoptosis as well as autophagy. Cells were pretreated with 3MA for 24 h, and were further exposed to TNF-α in the presence of the CK2 inhibitor DRB for 24 h. 3MA, 3-methyladenine. (A) A western blot assay showed that 3MA suppressed the conversion of LC3 from LC-3I to LC-3II. Confocal microscopy also demonstrated that 3MA suppressed the appearance of a punctuate LC3 pattern. The graph showing the quantification data of the fraction of cells with puncta supported that 3MA significantly suppressed puncta formation (** *P*<0.01). β-actin, a loading control. (B) A viability assay showed that 3MA significantly protected chondrocytes against cell death (** *P*<0.01). (C) Flow cytometry demonstrated that 3MA prevented the accumulation of subdiploid cells. (D) A western blot assay showed that 3MA protected chondrocytes against the activation of caspase-2L, -8, and -3. See Figure 2 for other definitions.

**Figure 7:**
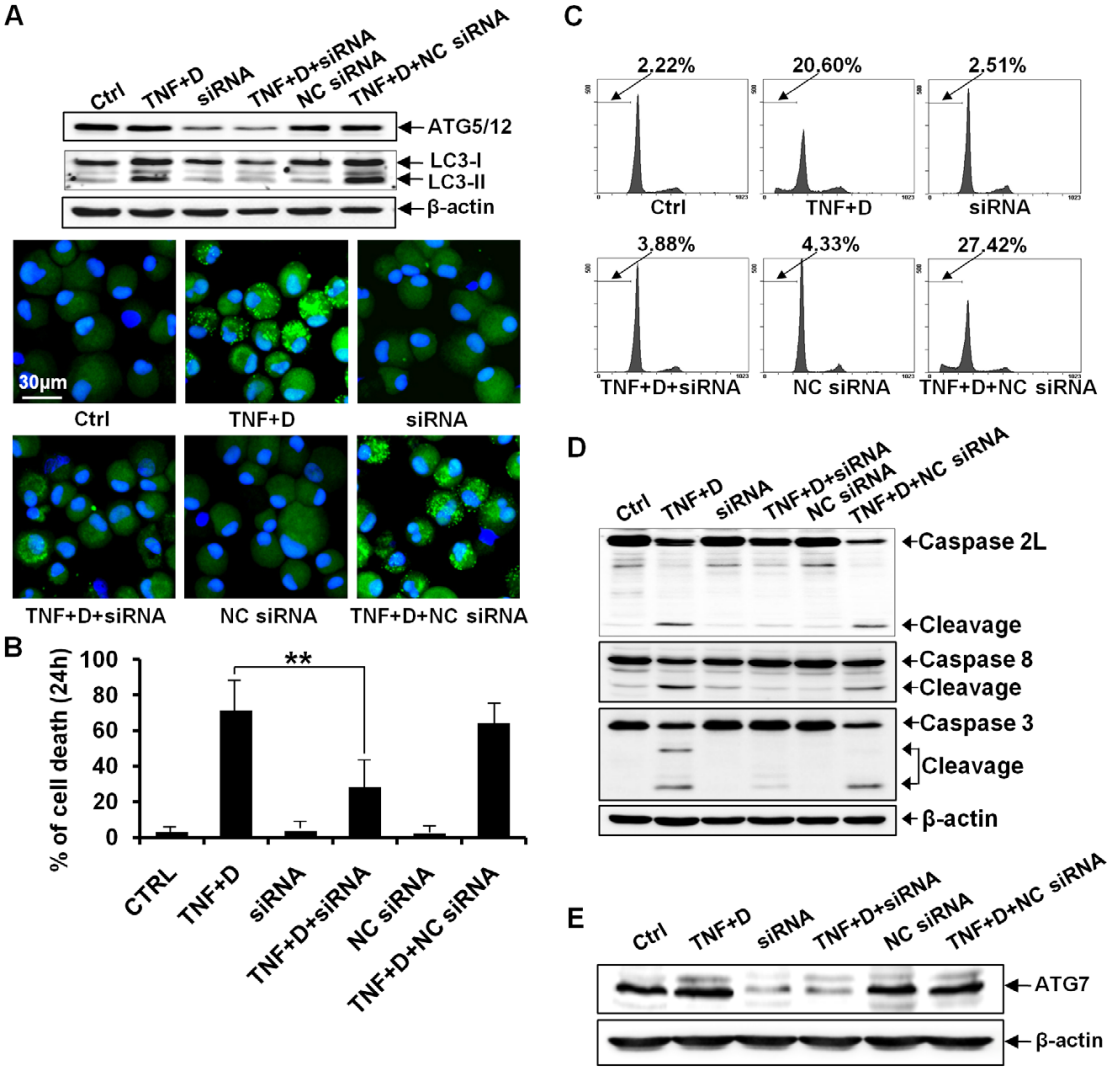
siRNA against ATG-5 protects chondrocytes against apoptosis as well as autophagy. For following assays, cells were harvested 24 h after treatment. NC siRNA, RNAi negative control; β-actin, a loading control. (A) A western blot assay showed that siRNA against ATG-5 efficiently reduced the expression level of ATG-5 protein and suppressed the conversion of LC3 from LC3-I to LC3-II. Confocal microscopy indicated that siRNA against ATG-5 suppressed the appearance of a punctuate LC3 pattern in chondrocytes co-treated with DRB and TNF-α. (B) A viability assay showed that siRNA against ATG-5 significantly protected chondrocytes against cell death (** *P*<0.01). (C) Flow cytometry demonstrated that siRNA against ATG-5 prevented the accumulation of subdiploid cells. (D) A western blot assay showed that siRNA against ATG-5 protected chondrocytes against the activation of caspase-2L, -8 and -3. (E) A western blot assay showing that siRNA against ATG-7 efficiently reduced the expression level of ATG-7 protein. See Figure 2 for other definitions.

We next tested whether pharmacological inhibition of autophagy by 3MA has impacts on the autophagic events of chondrocytes co-treated with DRB and TNF-α. Considering that 3MA is toxic to cells to some extent, we treated cells with 1 mM 3MA, the dose at which 3 MA did not yield any significant cellular damage after three days in culture. A western blot assay showed that 3MA suppressed the conversion of LC3 from LC3-I to LC3-II. Confocal microscopic observation and quantification assay revealed that 3MA suppressed the appearance of a punctuate LC3 pattern in chondrocytes co-treated with DRB and TNF-α (Fig. 6A). These data indicate 3MA efficiently protected chondrocyte against autophagy. We next investigated whether inhibition of autophagy by 3MA affects the induction of apoptosis in chondrocytes co-treated with DRB and TNF-α. Our viability assay showed that 3MA protected chondrocytes against cell death (Fig. 6B). Flow cytometry demonstrated that 3MA prevented the accumulation of subdiploid cells (Fig. 6C). Moreover 3MA protected chondrocytes against the activation of caspase-2L, -8, and -3 by the co-treament with DRB and TNF-α (Fig. 6D). These findings suggest that the inhibition of autophagy by 3 MA prevents the activation of caspase subtypes and results in the protection of cells against apoptosis. The inhibition of autophagy was also carried out by knock-down of ATG-5 and ATG-7 genes. A western blot assay showed that siRNA against ATG-5 efficiently reduced the expression level of ATG-5 protein and that siRNA against ATG-5 efficiently suppressed the conversion of LC3 from LC3-I to LC3-II. Confocal microscopic observation revealed that siRNA against ATG-5 suppressed the appearance of a punctuate LC3 pattern in chondrocytes co-treated with DRB and TNF-α (Fig. 7A). Viability assay showed that siRNA against ATG-5 protected chondrocytes against cell death (Fig. 7B). Flow cytometry demonstrated that siRNA against ATG-5 prevented the accumulation of subdiploid cells (Fig. 7C). Furthermore, siRNA against ATG-5 protected chondrocytes against the activation of caspase-2L, -8, and -3 by the co-treament with DRB and TNF-α (Fig. 7D). siRNA against ATG-7 also efficiently reduced the expression level of ATG-7 protein (Fig. 7E) and protected chondrocytes against apoptosis as well as autophagy (data not shown). These findings suggest that increased autophagosome formation is required for the induction of apoptosis in articular chondrocytes.

### LC3-II-positive autophagic cells were detected in cartilage obtained from OA model rats and human OA patients

**Figure 8:**
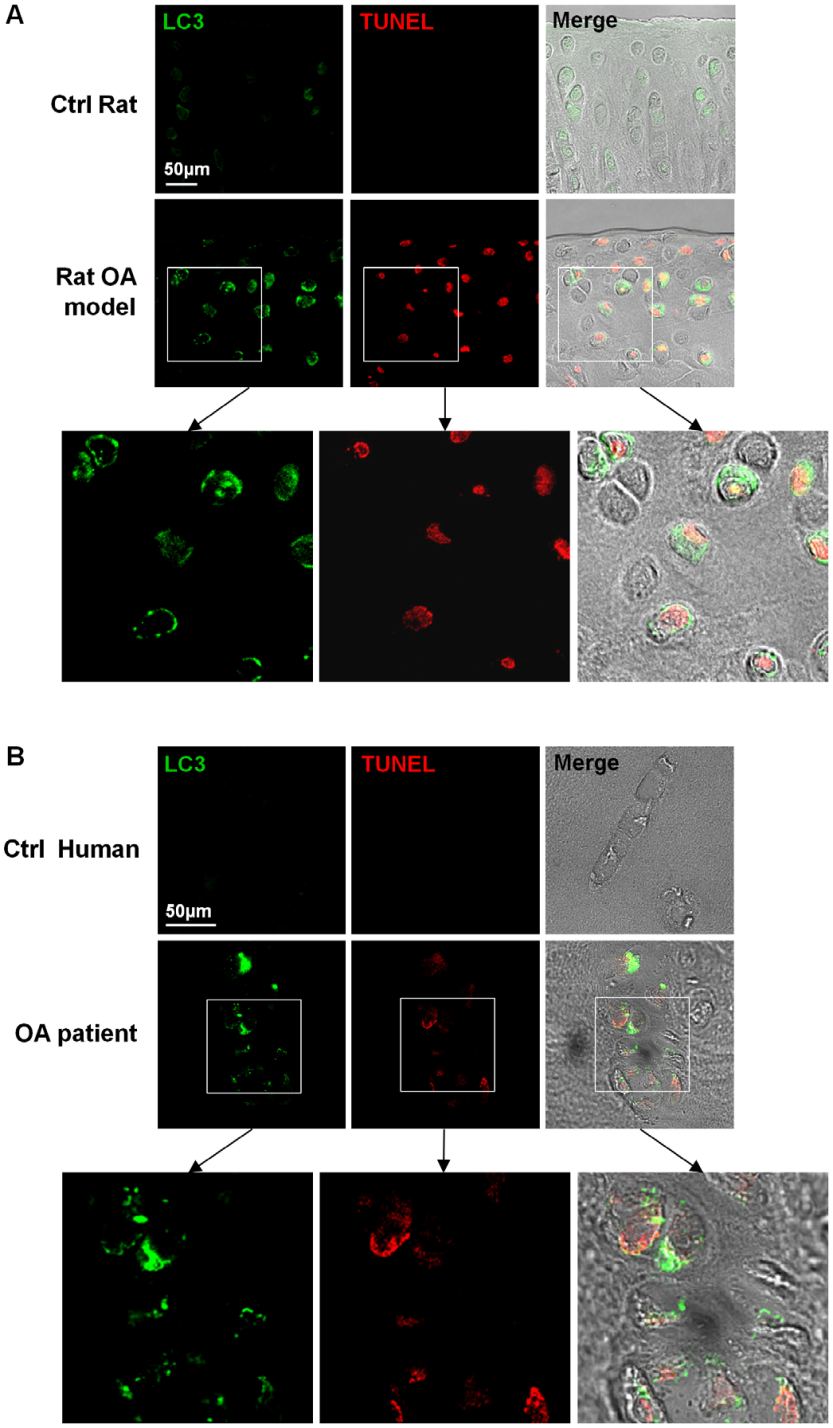
Demonstration of LC3-II positive punctuate autophagic cells in cartilage obtained from OA model rats and human OA patients. Fluorescent images were observed and photographed using a laser-scanning confocal microscope under DIC optics without counterstaing. (A) Chondrocytes in cartilage obtained from OA model rats display a punctuate LC3 pattern. Several cells showing autophagic LC3 puncta are TUNEL positive. (B) Chondrocytes from cartilage obtained from human OA patients display a punctuate LC3 pattern. Several cells showing autophagic LC3 puncta are TUNEL positive. Representative data are shown.

Finally, we examined whether autophagy was involved in OA pathogenesis. Compared to the control rats, the expression level of LC3 was higher in chondrocytes of the cartilage obtained from OA model rats (Fig. 8A). Noticeably, chondrocytes of OA model rats showed a punctuate LC3 pattern. However, chondrocytes of the cartilage obtained from control rats displayed diffuse LC3 staining. Among 9 animals tested, 7 showed LC3-II-positive cells. We observed a punctuate LC3 pattern in TUNEL positive cells (Fig. 8A). Chondrocytes of the cartilage obtained from human OA patients also showed a punctuate LC3 pattern (Fig. 8B). Among 20 samples tested, 12 showed LC3-II-positive cells. The expression level of LC3 in chondrocytes of the cartilage obtained from control human was so low that not even diffuse LC3 staining was observable. We observed a punctuate LC3 pattern in TUNEL positive cells (Fig. 8B). Approximately 30% of cells showing LC3 punta were TUNEL positive in both experimental groups. To this end, we examined whether autophagic and apoptotic events increase in rat articular cartilage chondrocytes with advancing age. Expression levels of LC3-II and caspase-3 cleavage product in the articular chondrocytes of 21- and 30-month-old rats were increased compared to their 6-month-old counterparts (Figure S4).

## Discussion

The present study elucidated that CK2 activity in articular chondrocytes is decreased in aged rats compared to young controls and that downregulation of CK2 activity facilitates TNF-α-mediated chondrocyte death. Although several previous reports showed that CK2 modulates TNF-α mediated cell death [29]-[31], the modulation of TNF-α-mediated chondrocyte death by CK2 has not been well studied. Because TNF-α is a major factor inducing chondrocyte death, we hypothesized that the downregulation of CK2 activity in aged articular chondrocytes is involved in OA pathogenesis.

Despite the large number of studies in this field, controversies regarding the definition of cell death and the classification of cell death types have not yet been resolved. Cell death can be classified according to morphological appearance, enzymological criteria, functional aspects or immunological characteristics. Based on morphological criteria, three types of cell death can be defined: apoptosis, autophagy and necrosis. However, several critiques have been raised against the clear-cut distinctions of these three types of cell death. To date, a clear equivalence between ultrastructural alterations and biochemical cell death characteristics has not been established [32], [33].

Autophagy has been used to describe the catabolic pathways of the degradation of intracellular macromolecules. Autophagy starts with the sequestration of cytoplasmic organelles in a membrane vacuole called an autophagosome. Next, autophagosomes fuse with lysosomes, in which cellular materials are degraded and recycled. Because numerous recent studies have shown that increased autophagic activity is associated with cell death [34]-[36], autophagy is now considered to be a type of cell death. Autophagic cell death is accompanied by massive autophagic vacuolation. Autophagic cell death involves the caspase-dependent mechanism in some contexts but not in others [37], [38]. Although the involvement of autophagy in cell death has been elucidated in multiple experimental and physiological settings, the role of autophagy in dying cells remains a subject of debate [39], [40]. Autophagy can exert both cytoprotective and cell death functions, depending on the specific cellular conditions. On one hand, autophagy can promote the survival of dying cells in the absence of apoptosis [41], and the inhibition of autophagy triggers apoptotic cell death [42], [43]. On the other hand, in certain settings, the inhibition of autophagy at an early stage can prevent the induction of apoptosis [44].

Arguably, the most intriguing finding of our study is the participation of autophagy in chondrocyte death. To date, little progress has been made in understanding the participation of autophagy in the regulation of chondrocyte death. Most previous studies of chondrocyte autophagy had examined the fate of growth plate chondrocytes [45]–[48]. These studies demonstrated that autophagy plays a cyto-protective role in chondrocytes and that the suppression of autophagy leads to elevated cell death [46], [47]. However, in other settings, 3MA-treated chondrocytes became refractory to cell death stimuli, suggesting that sustained autophagy promotes cell death [45]. Uncoupling protein 3, PIM-2 (proviral integration of Moloney virus) and hypoxia-inducible factor 1 and 2 are known to regulate chondrocyte autophagy [45]–[47], [49], [50]. In addition, those studies showed that autophagy undergoes cross-talk with apoptosis via classical apoptotic mediators such as BID and Bad [46], [47]. Aside from these studies, little information exists concerning the molecular mechanisms underlying the regulation of chondrocyte apoptosis by autophagy.

In the present study, we revealed the findings supporting the induction of autophagy, as well as apoptosis, in chondrocytes co-treated with DRB and TNF-α. Importantly, the inhibition of autophagy by 3MA or siRNA against ATG-5 and -7 prevented the activation of caspase subtypes and protected chondrocytes against apoptosis. Although our data do not exclude the possibility that autophagy and apoptosis may contribute to cell death independently, autophagy, at least in part, leads to the induction of apoptosis in chondrocytes co-treated with DRB and TNF-α. The presence of this cross-talk between apoptosis and autophagy is additionally supported by EM data, which show numerous vacuoles in cells with apoptotic nuclei. The appearance of a punctuate LC3 pattern in apoptotic cells that show positive TUNEL staining also supports the presence of this cross-talk. The present study suggests that autophagy can participate in OA pathogenesis. Since most previous efforts to understand the detailed mechanism of chondrocyte death pertaining to OA pathogenesis have been devoted to the study of apoptosis [3], [9], [22], much less is known currently about the involvement of autophagy in OA pathogenesis. A report demonstrated that, with aging and the onset of osteoarthritis, the subsequent lowered expression of HIF-2α causes an increase of chondrocyte autophagy and the autophagic activity of chondrocytes, resulting in sensitization to apoptogen challenges [45]. Another report elucidated that high incidence of active caspase 3 as well as LC3-II expression are observed in the same cell of the superficial and middle zones of articular cartilage, indicating that the degenerations of cartiage results from a combination of apoptosis and autophagy [51]. In the present study, we observed that downregulation of CK2 activity facilitated TNF-α-mediated chondrocyte death through autophagy *in vitro.* Furthermore, we observed autophagic cells in cartilage obtained from OA model rats and human OA patients. The present study with the two previous reports [45], [51] suggests that autophagy participates in OA pathogenesis although it should be clarified in the future if autophagy observed is a consequence versus a cause of the degeneration *in vivo* Conversely, a previous study reported that autophagy is a protective mechanism in normal cartilage, and its aging-related loss is linked with cell death and osteoarthritis [52]. These opposing findings suggest that functional relationship between apoptosis and autophagy during OA pathogenesis appears to be complex. While autophagy probably could be activated as an adaptive response to avoid cell death, this process also appears to be conjunctly activated with apoptosis. Although these reports mutually contradictory, they suggest the possibility that compromised autophagy may contribute to the development of OA. Thus, the potential role of autophagy in the development of osteoarthritis should be further examined in the future.

Another interesting finding in the present study is the involvement of CK2 in chondrocyte autophagy. CK2 is well known as a major kinase participant in apoptosis regulation. The regulation of apoptosis by CK2 has been studied extensively. However, understanding the regulation of autophagy by CK2 is fragmentary. Although we speculate that downregulation of CK2 activity modulates the molecules involved in the autophagy pathway, the signaling pathway by which CK2 regulates autophagy remains to be determined.

Because cell death mechanisms play a role in OA pathogenesis, modulation of these mechanisms may have substantial therapeutic potential. The data presented here underline the importance of the treatment approaches of targeting apoptosis and autophagy for OA. Considering that these approaches should target both apoptotic and autophagic pathways, deciphering the detailed molecular mechanism by which CK2 modulates chondrocyte death via multiple pathways is an important and challenging task.

Taken together, we conclude that downregulation of CK2 activity facilitates TNF-α-mediated chondrocyte death through apoptosis and autophagy.

## Supporting Information

Figure S1 **Slight reduction in chondrocyte viability by CK2 inhibitors.** Cells were treated with one of three CK2 inhibitors (apigenin, DRB or TBB) for 24 h. Viability was determined by a cell counter performing an automated trypan blue exclusion assay. Treatment with two CK2 inhibitors (apigenin and DRB) at a concentration of 75 and 100 μM led to a slight reduction in chondrocyte viability.
(TIF)

Figure S2 **Facilitation of TNF-α-mediated chondrocyte death by apigenin via apoptosis.** In addition to DRB, apigenin (100 μM) facilitated TNF-α-mediated chondrocyte death via apoptosis. The facilitation of the activation of caspase subtypes by apigenin is presented.
(TIF)

Figure S3 **Effects of CK2α knockdown and overexpression on TNF-α-mediated chondrocyte death.** (A) TNF-α-mediated chondrocyte death was significantly facilitated by silencing of CK2α by siRNA (** *P*<0.01). CK2 stealth siRNA were as follow: sense, 5′-CAA ACU AUA AUC GUA CAU C-3′; antisense, 5′-GAU GUA CGA UUA UAG UUU G -3′. As a negative control, stealth RNAi negative control (Invitrogen) was used. Transfection procedure is described in Materials and Methods. Cells were further exposed to 50 ng/ml TNF-α for 24 h. NC siRNA, RNAi negative control. (B) TNF-α-mediated chondrocyte death was significantly reduced by overexpression of CK2α (** *P*<0.01). Full-length CK2α was cloned by amplifying the rat cDNA with primers 5′-ATAGAATTCATGTCGGGACCCGTGCCAAGCAG-3′ (*EcoRI* site underlined) and 5-GCATCTAGATTACTGCTGAGCGCCAGCGG-3′ (*XabI* site underlined). The PCR product was subcloned into the mammalian expression vector pcDNA3. Transfection procedure is described in Materials and Methods. Cells were further exposed with 50 ng/ml TNF-α in parallel with 100 μM DRB for 24 h. Ctrl, untransfected control cells. Vec, cells tranfected with pcDNA3 vector.
(TIF)

Figure S4 **Autophagic and apoptotic events increase in rat articular cartilage chondrocytes with advancing age.** Articular chondroctyes obtained from 6-, 21- and 30-month-old rats were used for western blot assay. (A) A western blot showing that expression levels of LC3-II of 21- and 30-month-old rats were increased compared to their 6-month-old counterpart. (B) A western blot showing that expression levels of caspase-3 cleavage product in the articular chondrocytes of 21- and 30-month-old rats was increased compared to their 6-month-old counterpart.
(TIF)
