# Supplementary figures and images for: Downregulation of Protein Kinase CK2 Activity Facilitates Tumor Necrosis Factor-α-Mediated Chondrocyte Death through Apoptosis and Autophagy
Source: PLoS One. 2011 Apr 29;6(4):e19163. doi: 10.1371/journal.pone.0019163 (PMC3084779; doi:10.1371/journal.pone.0019163)

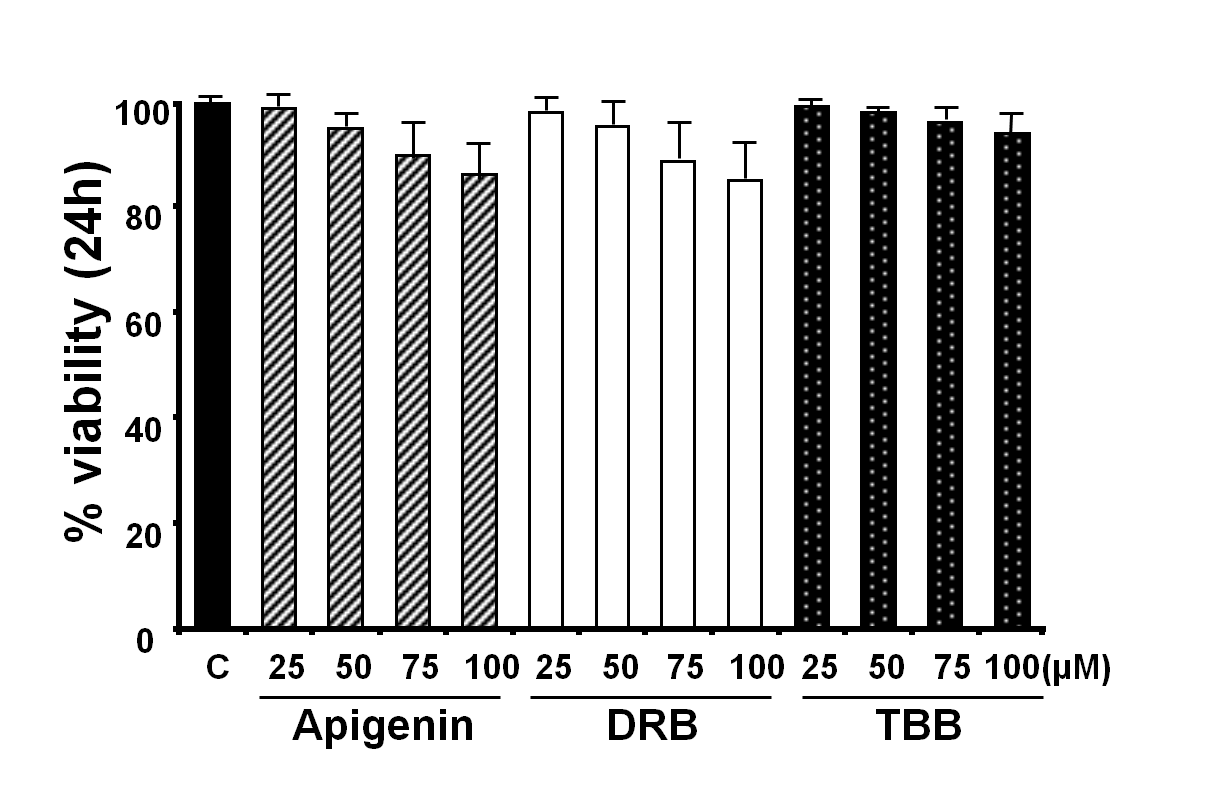

Supplement: Figure S1 — Slight reduction in chondrocyte viability by CK2 inhibitors. Cells were treated with one of three CK2 inhibitors (apigenin, DRB or TBB) for 24 h. Viability was determined by a cell counter performing an automated trypan blue exclusion assay. Treatment with two CK2 inhibitors (apigenin and DRB) at a concentration of 75 and 100 μM led to a slight reduction in chondrocyte viability. (TIF) [file pone.0019163.s001.tif]

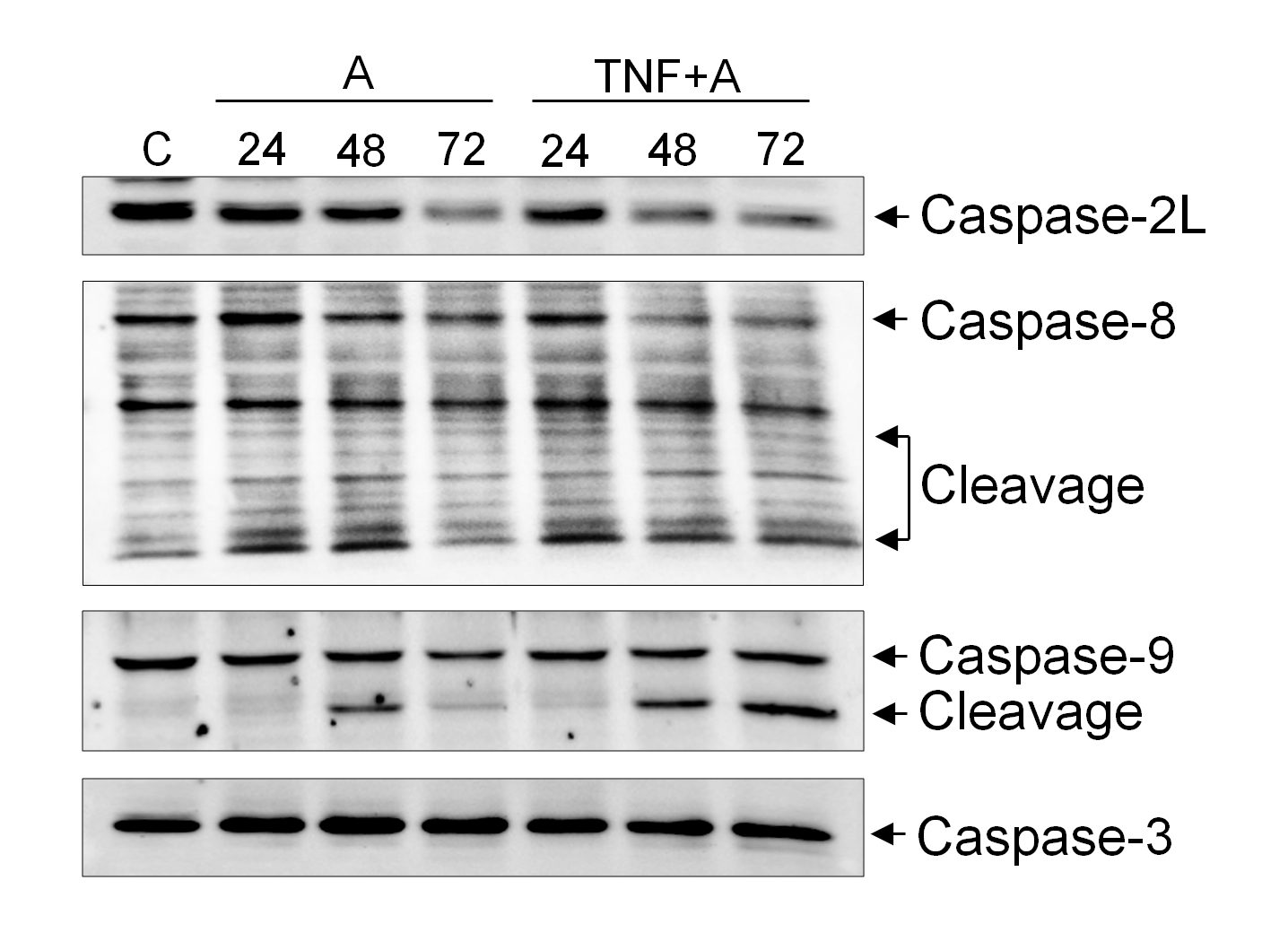

Supplement: Figure S2 — Facilitation of TNF-α-mediated chondrocyte death by apigenin via apoptosis. In addition to DRB, apigenin (100 μM) facilitated TNF-α-mediated chondrocyte death via apoptosis. The facilitation of the activation of caspase subtypes by apigenin is presented. (TIF) [file pone.0019163.s002.tif]

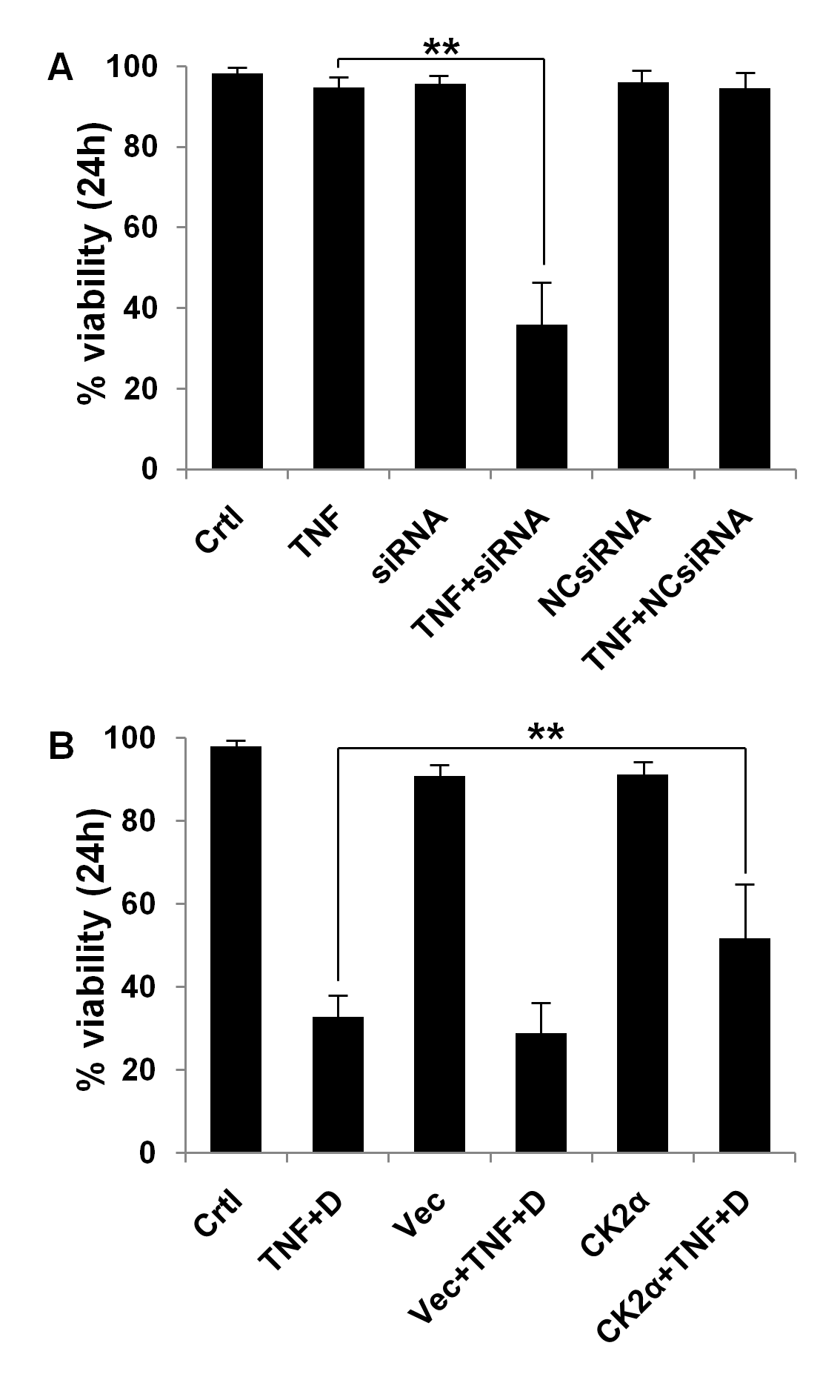

Supplement: Figure S3 — Effects of CK2α knockdown and overexpression on TNF-α-mediated chondrocyte death. (A) TNF-α-mediated chondrocyte death was significantly facilitated by silencing of CK2α by siRNA (** P<0.01). CK2 stealth siRNA were as follow: sense, 5′-CAA ACU AUA AUC GUA CAU C-3′; antisense, 5′-GAU GUA CGA UUA UAG UUU G -3′. As a negative control, stealth RNAi negative control (Invitrogen) was used. Transfection procedure is described in Materials and Methods. Cells were further exposed to 50 ng/ml TNF-α for 24 h. NC siRNA, RNAi negative control. (B) TNF-α-mediated chondrocyte death was significantly reduced by overexpression of CK2α (** P<0.01). Full-length CK2α was cloned by amplifying the rat cDNA with primers 5′-ATAGAATTCATGTCGGGACCCGTGCCAAGCAG-3′ (EcoRI site underlined) and 5-GCATCTAGATTACTGCTGAGCGCCAGCGG-3′ (XabI site underlined). The PCR product was subcloned into the mammalian expression vector pcDNA3. Transfection procedure is described in Materials and Methods. Cells were further exposed with 50 ng/ml TNF-α in parallel with 100 μM DRB for 24 h. Ctrl, untransfected control cells. Vec, cells tranfected with pcDNA3 vector. (TIF) [file pone.0019163.s003.tif]

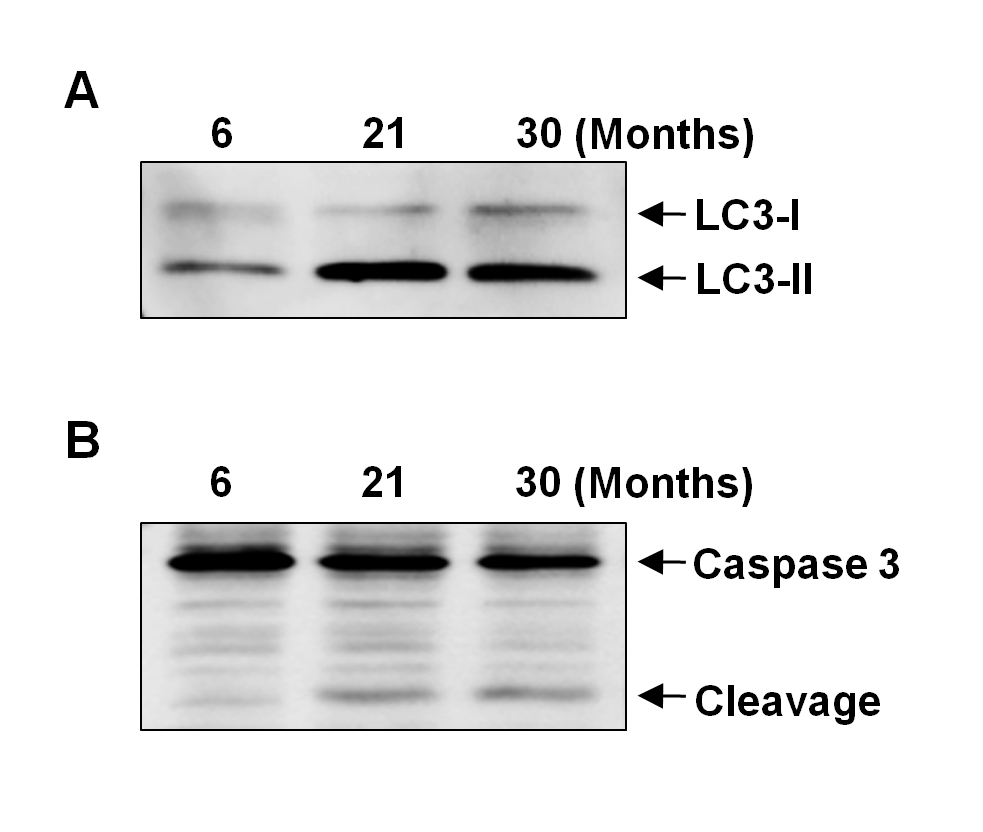

Supplement: Figure S4 — Autophagic and apoptotic events increase in rat articular cartilage chondrocytes with advancing age. Articular chondroctyes obtained from 6-, 21- and 30-month-old rats were used for western blot assay. (A) A western blot showing that expression levels of LC3-II of 21- and 30-month-old rats were increased compared to their 6-month-old counterpart. (B) A western blot showing that expression levels of caspase-3 cleavage product in the articular chondrocytes of 21- and 30-month-old rats was increased compared to their 6-month-old counterpart. (TIF) [file pone.0019163.s004.tif]
